# Differential motor neuron activity in rats during successful and failed grasping

**DOI:** 10.1093/cercor/bhaf032

**Published:** 2025-03-02

**Authors:** Riccardo Viaro, Davide Bernardi, Emma Maggiolini, Alessandro D’Ausilio, Carolina Giulia Ferroni, Pierantonio Parmiani, Luciano Fadiga

**Affiliations:** Section of Physiology, Department of Neuroscience and Rehabilitation, University of Ferrara, Ferrara 44121, Italy; Center for Translational Neurophysiology, Istituto Italiano di Tecnologia, Ferrara 44121, Italy; Center for Translational Neurophysiology, Istituto Italiano di Tecnologia, Ferrara 44121, Italy; Department of Physics and Astronomy, University of Padova, Padova 35131, Italy; Section of Physiology, Department of Neuroscience and Rehabilitation, University of Ferrara, Ferrara 44121, Italy; Section of Physiology, Department of Neuroscience and Rehabilitation, University of Ferrara, Ferrara 44121, Italy; Center for Translational Neurophysiology, Istituto Italiano di Tecnologia, Ferrara 44121, Italy; Center for Translational Neurophysiology, Istituto Italiano di Tecnologia, Ferrara 44121, Italy; Section of Physiology, Department of Neuroscience and Rehabilitation, University of Ferrara, Ferrara 44121, Italy; Section of Physiology, Department of Neuroscience and Rehabilitation, University of Ferrara, Ferrara 44121, Italy; Center for Translational Neurophysiology, Istituto Italiano di Tecnologia, Ferrara 44121, Italy

**Keywords:** error-signal, M1, intracortical microstimulation, prehension, single-neuron recording

## Abstract

A substantial body of literature has focused on neural signals evoked by errors emerging during the execution of goal-directed actions. It is still unclear how motor cortex activity during movement execution relates to feedback error processing. To investigate this, we recorded primary motor cortex (M1) single-unit activity in rats during a grasping task. About half of the recorded neurons showed modulation of their firing activity that did not depend on success or failure, which we termed outcome-independent neurons. Other neurons showed a difference in their discharge profile when comparing successful and unsuccessful trials, which we called outcome-dependent neurons. Among both outcome-dependent and -independent neurons, we further distinguished neurons presenting their maximum firing rate in specific epochs as defined by the task. We compared the cortical distribution of outcome-independent and outcome-dependent neurons to cortical maps of complex forelimb movements evoked by intracortical microstimulation in additional animals. The majority of outcome-independent neurons was localized within the limb extension and paw open-closure movement representations. Outcome-dependent neurons were not clearly associated to particular motor representations. Cortical arrangement of neurons, both outcome-independent and outcome-dependent, and their correlation with distinct movement representations, can serve as indicator for anticipating potential outcomes before the conclusion of an action.

## Introduction

In the course of daily activities, occasional movement-related errors are inevitable, often occurring without significant behavioral consequences. Nevertheless, these errors are an essential component of motor learning (Deng et al. 2023). An error signal can emerge from explicit external feedback (Goodale et al. 1986; Carson et al. 1993; Khan et al. 2004; Krigolson and Heath 2004; Heath 2005; Khan and Lawrence 2005), as well as through self-action monitoring (Körding and Wolpert 2004; Desmurget and Grafton 2000; Wolpert and Ghahramani 2000; Shadmehr et al. 2010). A substantial body of literature has focused on the neural correlates of feedback error processing (Holroyd and Coles 2002; Hoffmann and Beste 2015). The different aspects of the error are processed in distinct neural circuits that operate on different timescales (Gabitov et al. 2020). Error-specific signals arise in an extended neural network that includes the anterior cingulate cortex (ACC; Carter et al. 1998; Holroyd et al. 2004, Alexander and Brown 2011; Vassena et al. 2020), basal ganglia (Bech et al. 2023) and cerebellum (Thach et al. 1992; Blakemore et al. 2001; Maschke et al. 2004; Smith and Shadmehr 2005; Xu-Wilson et al. 2009; Criscimagna-Hemminger et al. 2010; Hanajima et al. 2015).

In addition to this brain network, single neuron activity in the primary motor cortex (M1) can also be modulated by errors occurring towards the end of a goal-directed action (Krigolson and Holroyd 2007; Krigolson et al. 2008; Milekovic et al. 2012, 2013; Bonini et al. 2014; Inoue et al. 2016; Even et al. 2017; Levy et al. 2020). Indeed, M1 receives inputs from several cortical and subcortical structures involved in error-processing, including dopaminergic projections (Luft and Schwarz 2009), input from somatosensory areas, and indirect input from the cerebellum. Furthermore, M1 and premotor cortices encode end-point errors during a reaching task (Inoue et al. 2016). In mice performing a repeated task, the initial state of layer V M1 neurons contains a memory trace of the previous trial’s outcome, a state probably associated with the feedback activity of layer II-III neurons (Levy et al. 2020). However, while M1 pyramidal neurons in layer V certainly play a key role in the control of movements (Beloozerova et al. 2003), it remains unclear whether they merely reflect error processing happening in other areas or they contribute to error processing itself.

In the present study, we recorded layer V M1 neurons during correctly and incorrectly executed grasping movements. Using 32-channel intracortical arrays implanted in the forelimb motor representation of trained rats, we tested whether the discharge profile of grasping motor neurons differed during action execution, depending on whether the action ultimately succeeded or failed. Then, we investigated their cortical distribution in relation to a movement representation map obtained via a long-duration intracortical microstimulation (ICMS) procedure conducted in separate rats trained on the same task. This procedure was designed to assess whether the cortical arrangement of neurons modulated by action outcomes co-localized with specific movement representations. We demonstrate that outcome-dependent neurons are not localized within a specific region of the motor map probably reflecting a limited involvement in motor execution. Among these neurons, a subclass was modulated before the completion of an erroneous action, thus supporting an active contribution to the processing of motor errors.

## Materials and methods

### Animals

We used ten adult male Long-Evans rats (Charles River Laboratories, Calco, Italy), each weighing 250–300 g. Females were excluded from the study because cyclically fluctuating hormone levels can make behavioral data more variable and difficult to interpret. Specifically, we utilized 5 animals for single-neuron recording and 5 animals for long-duration ICMS. Throughout the experimental period, animals were individually housed in cages maintained under regular lighting conditions (12 hr light/dark cycle), as well as constant temperature (20°C–22°C) and humidity (55%–65%) ranges. All experimental sessions were conducted during the first part of the light period (08:00 a.m.–12:00 p.m.). The experimental protocol complied with the Animal Research Reporting of In Vivo Experiments (ARRIVE) guidelines and the European Communities Council Directive of 1986 November 24 (86/609/EEC). It was designed in compliance with the Italian law regarding the care and use of experimental animals (DL26/2014), and approved by the institutional review board of the University of Ferrara and the Italian Ministry of Health (permission n. 989/2020-PR). We took adequate measures to minimize animal pain as well as the number of animals, according to the three Rs principle (Russell and Burch 1959).

### Behavioral task

**Fig. 1:**
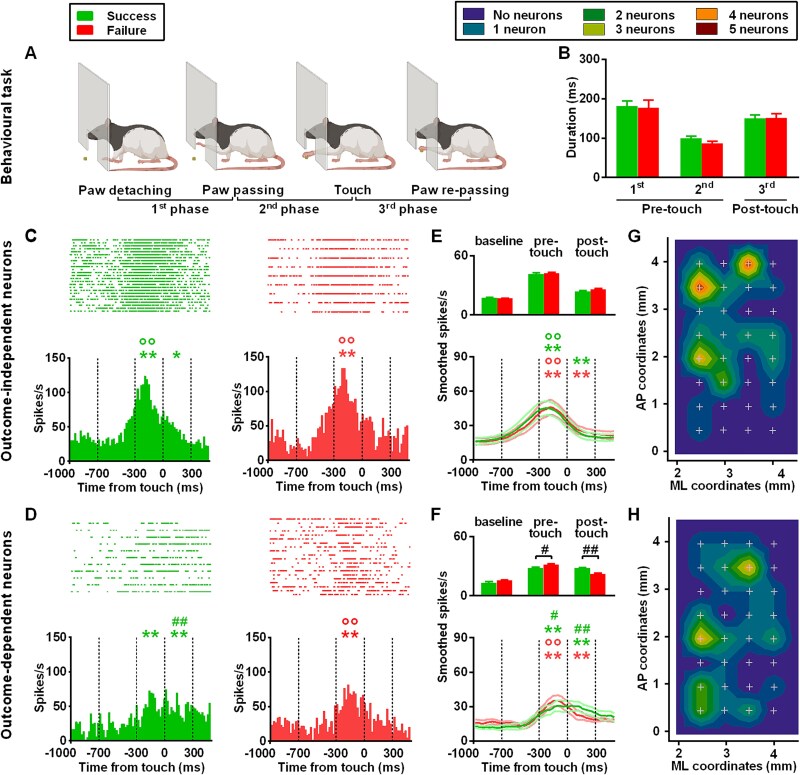
Behavioral task, discharge profile, and cortical maps of outcome-independent and outcome-dependent neurons. (A, B) Behavioral task reported using a schematic representation (A) and evaluated as time duration of the different phases (B). (C, D) Raster plots aligned to touch (top panels) and histograms (bottom panels) of a representative outcome-independent (C) and outcome-dependent (D) neuron during successful and failed grasping. (E,F) Smoothed averaged profiles (bottom panels) and histograms (top panels) of all neurons displaying the same modulation of those shown in panels C and D. Vertical dotted lines mark the three 300-ms epochs (baseline, pre- and post-touch). Errors bars and shade line represent SEM. (G,H) Cumulative cortical maps of outcome-independent (G) and outcome-dependent (H) neurons. In the frequency distribution maps, x and y values correspond to the coordinates relative to the bregma (ML and AP, respectively) and color coding corresponds to the cumulative frequency of neurons observed at each location. ^*^P < 0.05 and ^**^P < 0.01 difference from baseline, °°*P* < 0.01 difference from post-touch epoch, ^#^*P* < 0.05 and ^##^*P* < 0.01 difference from other outcome.

The overall apparatus was similar to that proposed by previous works (Viaro et al. 2017, 2021), and it consisted of a clear Plexiglas box, measuring 45 cm height × 15 cm width × 35 cm depth. We placed each rat in the box to perform a series of grasping tasks (Fig. 1A). We trained rats over a period of about 4 weeks, 5 days per week. The task required extending the preferred forelimb through an aperture measuring 15 cm in height and 1 cm in width to grasp and eat one rounded food pellet (~45 mg each; TestDiet, Richmond, USA), which was placed in a small indentation on a shelf located outside the slit which was 3 cm above the floor of the box. A commercial video camera (50 frames/s) positioned near the Plexiglas box provided the lateral view of the animal.

After an initial period of habituation (2–3 days), in which animals grasped with their mouth, we forced rats to use their paw, by withholding the food when they attempted to take it with their mouths. After a few days, rats developed a consistent preference for either the left or the right paw during the reaching/grasping phase, albeit they used both paws to bring the food to the mouth and hold it while eating (manipulation phase). An infrared sensor placed on the bottom of the indentation was triggered when the rat removed the food pellet. We also trained animals to walk to the rear wall of the box after each attempt, in order to readjust the body position before the next attempt. During each session, a rat performed about 40–50 attempts (usually ~ 20 min), with an inter-trial interval of 20–30 s. We considered a trial successful if a rat obtained the pellet and then consumed it, or if the pellet was dropped during retraction. We considered a trial as failed if the rat missed the pellet or pushed it out of the indentation due to imprecise movements. Failed trials occurred in an unpredictable sequence and in different proportions across the different sessions.

A rat completed the training when it performed at least 70% successful trials over five consecutive sessions. In order to maintain the vigilance and motivation high, we maintained rats on a restricted diet to keep their body weight at 90% of the normal free-feeding weight throughout experimental procedures. At the end of each session, we re-housed animals in their own cage, and we rewarded them with an extra dose of food pellets. Water was always available ad libitum.

### Anesthesia and surgery

After the end of training period, each rat underwent surgical procedures. Initially, we anesthetized rats with ketamine hydrochloride (80 mg/Kg, i.p.), and we used supplementary ketamine injections (4 mg/Kg, i.m. given as required, typically every 25–30 min) for the duration of the experimental session to maintain long-latency and sluggish hindlimb withdrawal upon pinching the hindfoot (stage III-1 and III-2; Friedberg et al. 1999; Tandon et al. 2008). We placed animals under anesthesia in a Kopf stereotaxic apparatus (David Kopf Instruments, Tujunga, USA), a heat lamp maintained the body temperature at 36°C–38°C. We performed a large craniotomy under stereomicroscopy (Carl Zeiss Meditec AG, S100/OPMI pico, Jena, Germany) to expose the frontal cortex of the hemisphere contralateral to the preferred limb (Viaro et al. 2011, 2014, 2017, 2021). Then, we differentiated procedures depending on whether we selected the animal for single-unit recording or long-duration ICMS mapping.

### Single-unit recording

We employed this procedure as described in a previous study, in which we evaluated the discharge of M1 forelimb neurons during a behavioral task in free-moving awake rats (Viaro et al. 2021). Single-unit recordings were performed in the hemisphere contralateral to the preferred limb. During surgery, we used a micromanipulator to lower vertically a 4 × 8 intracortical arrays (1.5 × 3.5 mm; MEA, Microprobes, Gaithersburg, USA) to a depth of 1500 μm below the cortical surface, corresponding to layer V of the frontal agranular cortex, in the area comprised between 2.5 and 4.0 mm in the mediolateral (ML) and 0.5 and 4 mm in the anteroposterior (AP) direction. This cortical region constitutes the forelimb motor representation (Viaro et al. 2011, 2014, 2017, 2021). The 32 active platinum-iridium electrodes (impedance: 0.5–1.5 MΩ at 1 KHz) had an inter-electrode distance of 0.5 mm in the ML and AP directions. Array dimensions ensure the recording of the entire forelimb motor cortex. A screw implanted in a more caudal position with respect to the craniotomy served as a reference. After array implantation, we covered the exposed cortex with silicone elastomer Kwik-Cast (Word Precision Instruments, Sarasota, USA), and we fixed the array to the skull using screws and dental cement (Jet Repair Acrylic; Lang Dental Manufacturing, Chicago, USA). Finally, we re-housed the rat in its cage and we treated it with analgesic (Ketorolac 5 mg/Kg i.p., daily) and antibiotic (Enrofloxacin 5 mg/Kg i.m., twice daily) drugs until the complete recovery of basal functions, such as gait balance and food-intake (usually two or three days).

After the recovery period, we conducted daily recording sessions (5 days a week) during the behavioral task, for up to 10 days, depending on the implant’s survival and the animal’s cooperation (Viaro et al. 2021). Before each recording session, we manually connected the implanted array to a wireless headstage interfaced with a receiver (W-Series, Triangle Biosystems International, Durham, USA) while gently holding the rats for a few seconds. The headstage amplified signals ×800 and bandpass filtered between 0.8 and 7000 Hz. Our software (Visual Basic. NET code and National Instrument library) further amplified (×10) and digitized signals with a sampling rate of 30 KHz. We detected and clustered off-line all waveforms with a stability through an entire session, using a band-pass filter (300–3000 Hz), a threshold of 3.5 standard deviations above and below the mean of the sample distribution, and a K-means Algorithm (Offline Sorter software; Plexon Inc, Greenville, USA).

### Long-duration ICMS

We used this procedure as in previous studies, in which we mapped complex forelimb movement representations in the ketamine-anesthetized rats (Bonazzi et al. 2013; Viaro et al. 2017). Long-duration ICMS was performed in the hemisphere contralateral to the preferred limb. Immediately after the surgical procedure, we changed the posture of the rat in the stereotaxic frame, letting its preferred forelimb hang freely, enabling movement against gravity in all directions. The resting position of each forelimb was in approximately half-way between extension and adduction and the wrist rested palm down with the digits slightly flexed. We penetrated the cortex using a glass-insulated tungsten microelectrode (0.6–1 MΩ, impedance at 1 kHz) mounted on an electrical microadvancer. Penetration sites were spaced over a 500 μm grid, with occasional adjustments to the coordinate grid to keep the electrode from penetrating a blood vessel. We did not report these adjustments in the reconstructed maps, because we did not perform the penetration at sites for which the required adjustment exceeded 50 μm. We lowered the electrode vertically to 1500 μm below the cortical surface, corresponding to layer V of the frontal agranular cortex (Viaro et al. 2011, 2014, 2017). The electrode delivered a 500-ms train of 200-μs bipolar pulses at 333 Hz. In each stimulation, we used biphasic current pulses, in which a positive phase followed a negative phase, to minimize damage that could occur during the stimulation (Graziano et al. 2002). Starting with a current of 10 μA, we increased the intensity in 5 μA steps until stimulus evoked an easily detectable multi-joint movement; then we adjusted the intensity to a level at which approximately 50% of the stimulation elicited movement. This level defined the current threshold, and two observers determined it. Occasionally, we observed some movement in the forelimb (or vibrissa) ipsilateral to the cortex, but the threshold current required to evoke it was higher than (almost double) the contralateral movement. Moreover, only negligible displacements characterized the ipsilateral-to-cortex movements. Once we detected a movement threshold (usually ~ 50 μA), we raised the current to 100 μA to increase the amplitude of movements and facilitate the quantitative characterization. If we detected no movement at 100 μA, we defined the site as non-responsive.

For the quantitative characterization of ICMS-evoked movements, we used an optical 3D motion capture system (Qualisys Motion Capture System; Qualisys North America Inc, Charlotte, USA) according to a previously described protocol (Bonazzi et al. 2013; Viaro et al. 2017). We positioned two adhesive infrared-reflective spherical markers (weight: 0.04 g, diameter: 0.30 cm) on the forelimb skin of animals at 2 anatomical landmarks, i.e. the dorsal middle of the wrist and the last-phalangeal joint of the 2 middle digits, to detect limb and paw movements, respectively. To minimize positional variability, the same operator positioned the markers in all experiments. Three infrared cameras positioned around the animals recorded the marker positions, using a 3D coordinate system, in which the x-, y-, and z-axes corresponded to the anterior, lateral and dorsal directions, respectively. The cameras recorded the movement for 2 s at a sampling rate of 100 Hz. We used the Qualisys Track Manager software for analyzing kinematic features off-line. We performed stimulation trials in the absence of spontaneous movements, and we did not modify the forelimb starting position. In addition, we performed sham stimulations without current delivery at two or three selected sites per animal by lowering the electrode vertically to 1500 μm below the cortical surface. During sham stimulations, we pressed the button used for delivering the stimulus, but the connection between the electrode and stimulus isolator unit had been previously interrupted using a switch. The absence of movement indicated that the current was responsible for a particular movement, and showed that there were no uncontrolled influences from set-up variability, such as noise generated by the operator or button pressing or incorrect current delivery. After sham stimulation, we also performed the actual stimulation at the 2 or 3 sham sites, which enabled us to rule out any further cause for a given movement other than cortical stimulation. Furthermore, although we tested rats under anesthesia, they were not motionless and inert, but instead displayed baseline physiological movements, namely a background of rhythmic thoracic expansion and contraction (breathing). Hence, in previous sets of experiments as well as during the sham stimulations, we recorded one or two trials (2 s each) per animal to measure marker displacement in the absence of electrode penetration and/or current delivery. This displacement due to background physiological movements at rest was 0.23 ± 0.10 mm. In order to unequivocally distinguish evoked from background movements, only movements exceeding a minimal displacement of the marker ≥1 mm on at least one of the x-, y-, or z-axes (i.e. movement cut-off) were used for data analysis. All measures were taken using the resting position of the marker as the origin of the Cartesian coordinate system. We expressed both the proximal and distal movements in terms of joint coordinates, and not end-point Cartesian coordinates.

We classified proximal forelimb movements according to the displacement of the wrist marker along the frontal (x), sagittal (y), and vertical (z) axes (Bonazzi et al. 2013; Viaro et al. 2017). Since we found a displacement on the x-, y-, and z-axis in all evoked limb movements, we defined several criteria to identify five movement classes. Abduction (Abd) and adduction (Add) movements present a maximal displacement on the y-axis involving a marker movement away from or towards the midline, respectively (Abd: y-axis positive > 4 mm and > 15% of x-axis, z-axis positive; Add: y-axis negative > 4 mm and > 15% of x-axis, z-axis positive). Extension (Ext) and retraction (Rtr) movements present a maximal displacement on the x-axis involving forward or backward movement of the marker, respectively (Ext: x-axis positive > 4 mm and > 15% of y-axis, z-axis positive; Rtr: x-axis negative > 4 mm and > 15% of y-axis, z-axis positive). Elevation movements (Elv) present a maximal displacement on the z-axis involving a marker movement upwards without a major shift on the x- and y-axis (z-axis positive > 4 mm and x- and y-axis < 4 mm). We obtained the paw component by subtracting the wrist marker value from the digit marker value at all points throughout its movement. We identified four distal movement classes, i.e. paw opening (Opn: x- and z-axis positive), paw closure (Clo: x-axis negative), opening/closure sequence (Ocs: x-axis positive followed by x-axis negative), and supination (Sup: z-axis positive). Simultaneous contraction of the digits characterized Opn, Clo, and Ocs, while Sup showed external rotation of the wrist without movement of the digits. Proximal and distal movements can appear alone or in combination (Bonazzi et al. 2013; Viaro et al. 2017). The visual inspection during stimulation provided additional information on the occurrence of the movements.

We obtained kinematic variables of each stimulation site by averaging the values recorded in 3 microstimulation trials. The time-point at which the tangential velocity exceeded 5% of maximum velocity (Adamovich et al. 2001) and the last time-point at which the marker reached maximal displacement on either the x-, y-, or z-axis define the onset and the end of movement, respectively. We did not take into consideration any displacement outlasting the stimulus and/or in the direction of the resting position.

### Electrode placement reconstruction

At the end of the single-unit recording sessions, we deeply anesthetized each animal with tiletamine/zolazepam hydrochloride (Zoletil 100, 10 mg/Kg i.m; Virbac Laboratories, Carros, France), to perform a perfusion transcardially with saline at room temperature, and a tissue’s fixation with cold 4% paraformaldehyde at pH 7.4. We removed brains, to make a post-fixation overnight, and to transfer the brain to 30% sucrose solution for cryoprotection, until they sunk. We used a freezing microtome (SM2000R; Leica Microsystems, Wetzlar, Germany) to cut 50 μm coronal sections, which we collected free floating in saline, for Nissl staining (Viaro et al. 2011, 2017, 2021). To do this, sections at the level of the motor cortex were mounted on chrome-alum-coated slides, stained with cresyl violet, dried in escalating alcohol concentration, cleared in xylene, coverslipped with mounting medium, and captured using a computer-interfaced light microscopy workstation (Zeiss Axioskop, Carl Zeiss, Jena, Germany) with a high-resolution digital camera (AxioCam ICc3, Carl Zeiss, Jena, Germany). We assessed the electrode placements and reconstructed them onto schematic templates of coronal sections (Paxinos and Watson 1982).

### Data presentation and statistical analysis

We defined a grasping neuron as a motor neuron significantly modulated by grasping execution. For each neuron, we subdivided spike trains recorded from each trial in two groups, based on action outcome, i.e. success or failure. We aligned spike trains with respect to when the food was touched (MATLAB Mathworks, Natick, USA). Then, we averaged spike trains over trials, and we binned them with a precision of 20 ms to obtain a time-dependent measure of the firing rate (spike/s; Fig. 1). To ensure that the task modulated the response of a selected neuron, we assessed differences in firing rate by one-way repeated-measures Analysis of Variance (ANOVA), with epoch as factor. We considered three different 300-ms epochs (Viaro et al. 2021), namely baseline (from 1000 to 700 ms before food touch), pre-touch (from 300 ms before food touch to food touch), and post-touch (from food touch to 300 ms after it). Based on previous works, we expected an early and later modulation (Viaro et al. 2021). We chose epoch duration based on the time it takes for the extension or retraction of the forelimb (usually ~ 250 ms each; Fig. 1B). Then we performed post-hoc analysis by Tukey’s test for multiple comparisons. We considered only neurons showing a significant increase in activity with respect to baseline. Due to the low sample size (*n* = 3), we excluded from the study neurons that decreased their basal activity during movement. The comparison between epochs-related activities of the two outcomes was assessed using a two-way ANOVA with outcome and epoch as factors. When the two-way repeated-measures ANOVA yielded significant factor interaction, we performed a post-hoc analysis using a Bonferroni test for multiple comparisons. Otherwise, if the two-way repeated-measures ANOVA was significant only at the factor level, we assessed differences for outcomes and epochs by Mann–Whitney tests. The single-neuron spike trains, aligned with respect to touch and averaged, were convolved with a Gaussian-Kernel function (width 15 bins) to obtain an appropriate smoothed spike density function, which provided a continuous time-dependent measure of firing patterns.

**Fig. 2:**
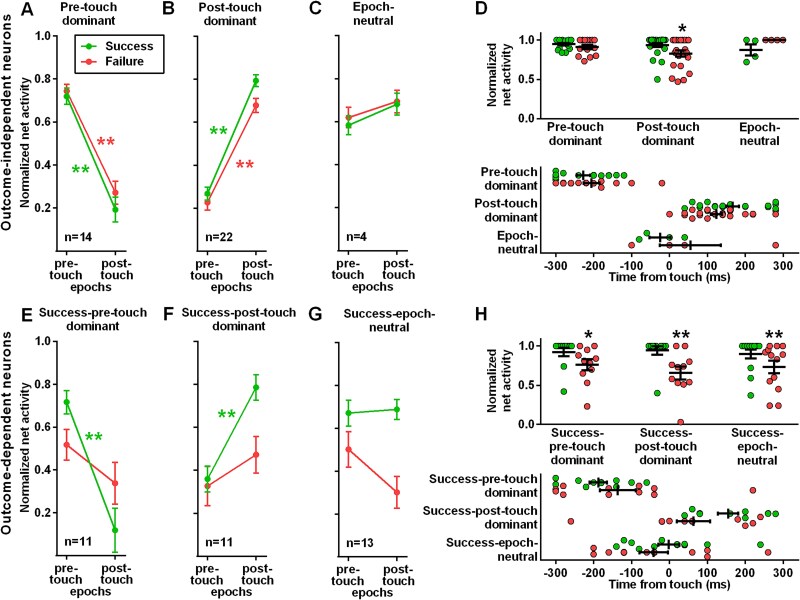
Activity of neuronal subpopulations in successful and failed grasping. (A-D) Neuronal subpopulations of the outcome-independent neurons. Normalized net activity of the pre-touch dominant (A), post-touch dominant (B) and epoch-neutral (C) neurons and their peak activity (D) as amplitude (top panel) and timing (bottom panel). (E-H) Neuronal subpopulations of the outcome-dependent neurons. Normalized net activity of the success-pre-touch dominant (E), success-post-touch dominant (F) and success-epoch-neutral (G) neurons and their peak activity (H) as amplitude (top panel) and timing (bottom panel). Data represent mean ± SEM. ^*^P < 0.05, ^**^P < 0.01 difference between pre-touch and post-touch epochs (A-C and E-G) or success and failure (D and H).

**Fig. 3:**
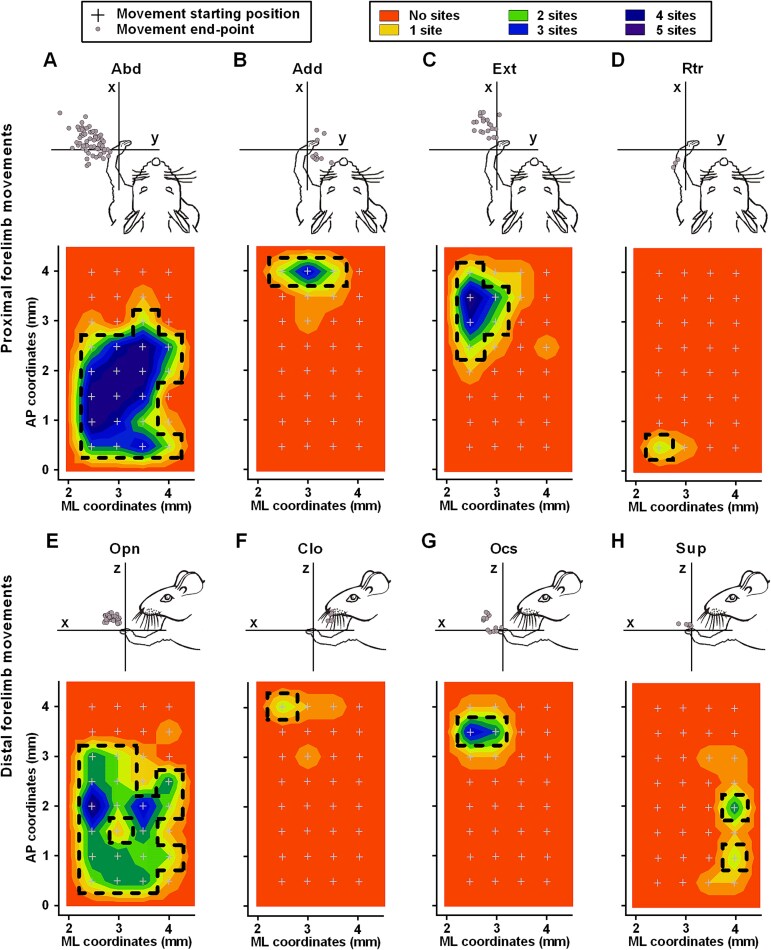
Cortical maps of complex forelimb movements evoked by long-duration ICMS. (A-D) Spatial end-points (top panels) and frequency distribution maps (bottom panels) of proximal forelimb movements, i.e. abduction (A), adduction (B), extension (C), and retraction (D). (E-H) Spatial end-points (top panels) and cumulative cortical maps (bottom panels) of distal forelimb movements, i.e. opening (E), closure (E), opening/closure sequence (G), and supination (H). In the top panels, the starting position (cross) and end-points of each movement (gray circles) are shown in the Cartesian axes that best identify the movement. In the frequency distribution maps, x and y values correspond to the coordinates relative to the bregma (ML and AP, respectively) and color coding corresponds to the cumulative frequency of movement sites observed at each location. Black dotted lines represent the borders of each movement representation, defined as the region in which the neuron density is larger than two stimulation sites per gridpoint. Note that end-points of opening/closure sequences form two clusters, each representing the opening and closure end-points of different phases in the same movement. Abbreviations: Abd, abduction; Add, adduction; Ext, extension; Rtr, retraction; Opn, opening; Clo, closure; Ocs, opening/closure sequence; Sup, supination.

To characterize the spatial distribution of recorded neurons and stimulation sites in the cortex, we constructed frequency maps averaged across animals, in a 2D bregma-relative spatially interpolated contour plot (Figs. 1-3). Specifically, we assigned to each recorded neuron or evoked-movement site an x- and y-value, corresponding to the mediolateral (ML) and anteroposterior (AP) coordinates relative to the bregma, respectively. We used color-coded maps to indicate the probability to find a neuron or site in a particular color-coded cortical region (Viaro et al. 2021). We defined each movement representation as the region encompassing the sites that evoked the movement in at least 2 of 5 animals. According to this definition, we found the topographical arrangement of movement sites to be consistent across animals (Bonazzi et al. 2013; Viaro et al. 2017).

For the behavioral analysis, we evaluated differences in the duration of the different task phases during success and failure by nonparametric Mann–Whitney tests (Fig. 1B).

To identify neuronal subpopulations, we classified each grasping neuron based on its discharge profile upon success and failure. In particular, we sub-classified neurons depending on whether the firing rate during 1 of the 2 touch-related epochs (pre- or post-touch) was significantly higher or comparable to the other touch-related epoch (Fig. 2). We did not consider categories composed of fewer than 4 neurons. We classified neurons based on their discharge profile upon success and failure, identifying 2 main populations: outcome-independent and outcome-dependent neurons. We then further divided each population into subpopulations, depending on the temporal discharge pattern. For outcome-independent neurons, which showed consistent discharge patterns regardless of the outcome, 3 subpopulations were defined. Pre-touch dominant neurons displayed maximum discharge in the pre-touch epoch. Similarly, post-touch dominant neurons exhibited maximum discharge in the post-touch epoch. Epoch-neutral neurons had similar firing activity in pre-touch and post-touch epochs. For outcome-dependent neurons, which varied their discharge patterns based on the trial outcome, 3 additional subpopulations were defined. In this case, to simplify the classification, we based on successful trials and we excluded information about activity in failed trials from the labeling. Success-pre-touch dominant neurons displayed maximum discharge in the pre-touch epoch during successful trials, regardless of their discharge patterns in failed trials. Success-post-touch dominant neurons exhibited maximum discharge in the post-touch epoch during successful trials, independently of their activity in failed trials. Lastly, success-epoch-neutral neurons had similar firing activity in pre-touch and post-touch epochs during successful trials, regardless of their discharge patterns in failure. Tables with the classification scheme are provided for reference (Tables 1, S1 and S2).

**Table 1:** Classification of grasping neurons based on factors influencing their discharge profile. The table presents populations (based on outcome modulation), subpopulations (based on temporal discharge profiles), the number of neurons, definitions, and net normalized activity values for the pre-touch and post-touch epochs during successful and failed grasping trials. Detailed statistical results are provided in the main text and Tables S1 and S2.

**Population**	**Subpopulation** **(n)**	**Definition**	**Pre-touch** **epoch:** **Success** **vs.** **Failure**	**Post-touch** **epoch:** **Success** **vs.** **Failure**
Outcome-independent neurons (*n* = 40)	Pre-touch dominant (*n* = 14)	Maximum activity in pre-touch epoch for both outcomes.	0.72 ± 0.04 vs. 0.74 ± 0.03	0.19 ± 0.06 vs. 0.27 ± 0.05
	Post-touch dominant (*n* = 22)	Maximum activity in post-touch epoch for both outcomes.	0.27 ± 0.03 vs. 0.23 ± 0.04	0.79 ± 0.03 vs. 0.68 ± 0.03
	Epoch-neutral (*n* = 4)	Similar activity in both epochs for both outcomes.	0.58 ± 0.04 vs. 0.62 ± 0.05	0.68 ± 0.05 vs. 0.69 ± 0.05
Outcome-dependent neurons (*n* = 38)	Success-pre-touch dominant (*n* = 11)	Maximum activity in pre-touch epoch for success, neutral response for failure.	0.72 ± 0.05 vs. 0.52 ± 0.07	0.12 ± 0.10 vs. 0.34 ± 0.10
	Success-post-touch dominant (n = 11)	Maximum activity in post-touch epoch for success, neutral response for failure.	0.36 ± 0.06 vs. 0.33 ± 0.09	0.79 ± 0.06 vs. 0.47 ± 0.09
	Success-epoch-neutral (*n* = 13)	Similar activity in both epochs for success, silent response for failure.	0.67 ± 0.06 vs. 0.50 ± 0.08	0.69 ± 0.05 vs. 0.30 ± 0.07

For the different neuronal subpopulations, we evaluated differences in the amplitude or timing of the peak activity during success and failure by nonparametric Mann–Whitney tests (Fig. 2).

**Fig. 4:**
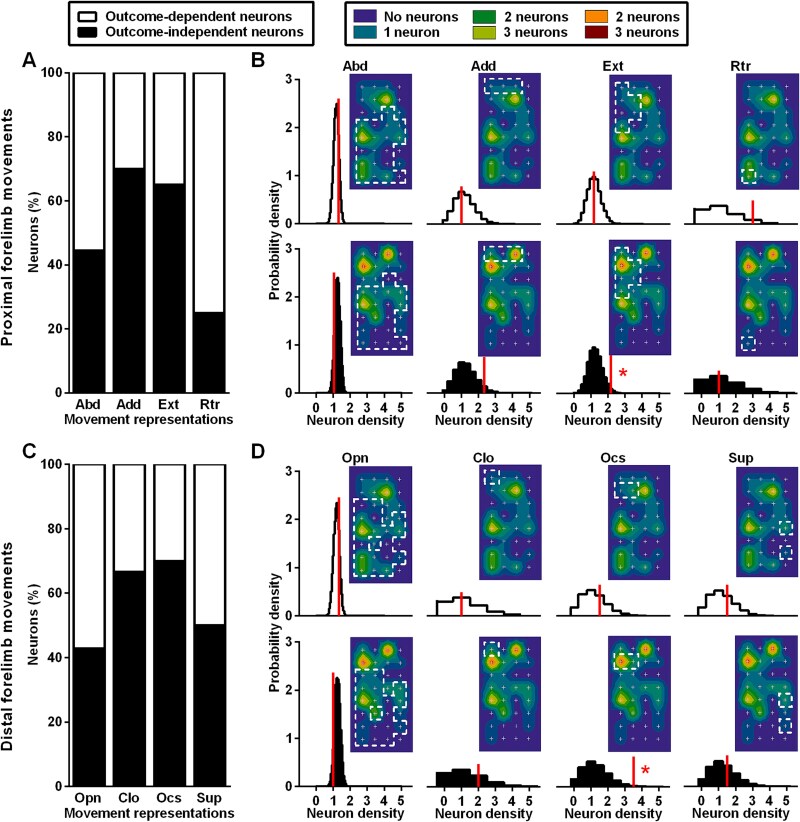
Proportion and density of outcome-independent and outcome-dependent neurons in the complex movement representations of the forelimb cortical maps. (A) Percent of outcome-independent and outcome-dependent neurons in the proximal forelimb movement representations. (B) Empirical probability density (normalized histogram) of outcome-dependent and outcome-independent neurons within each proximal movement representation. (C) Percent of outcome-independent and outcome-dependent neurons in the distal forelimb movement representations. (D) Empirical probability density (normalized histogram) of outcome-dependent and outcome-independent neurons within each distal movement representation. Vertical bars indicate the observed neuronal density. The null distribution was generated by randomizing neuron positions (see methods). Adjacent to each histogram, a color-coded map shows the cumulative cortical distribution of outcome-independent and outcome-dependent neurons (from Figs. 1G and H, respectively), overlaid with the borders of movement representations. These borders correspond to the frequency distribution maps for proximal and distal forelimb movements shown in Fig. 3A-D and E-H, respectively. The observed neuronal density reflects the overlap between the cortical map and movement representations, while the null distribution reflects the overlap based on randomized neuron positions abbreviations: Abd, abduction; Add, adduction; Ext, extension; Rtr, retraction; Opn, opening; Clo, closure; Ocs, opening/closure sequence; Sup, supination. ^*^P < 0.05 actual density different from null distribution.

To evaluate whether the fractions of outcome-independent and outcome-dependent neurons were different across ICMS-derived movement representations, we used a chi-square (χ^2^) test (Fig. 4). To achieve this, we overlaid the cumulative cortical maps of outcome-independent and outcome-dependent neurons (shown in Fig. 1G and H) with the frequency distribution maps of proximal and distal forelimb movements (presented in Fig. 3A–D and E–H, respectively). This overlay is explicitly displayed for each motor representation in the corresponding insets in Fig. 4B and D.

When the χ^2^ test resulted in a statistically significant *P*-value, we subsequently assessed the degree of dependence using Kramer’s V parameter. Furthermore, we wanted to assess whether the 2 classes of neurons, namely outcome-dependent and outcome-independent, exhibited overrepresentation within the subregions associated with specific movement representations (Fig. 4). To account for variations in region sizes, we constructed a null distribution using Monte Carlo simulations for each movement representation. Under the null hypothesis, we assumed that the neuron density (number of neurons per unit area) for both neuron types was uniformly distributed across the electrode grid. For each simulation, we assigned random positions on the grid to each neuron, and then calculated the number of neurons per unit area within each movement representation. Repeating this process generated an empirical null distribution. To determine statistical significance, we compared the observed neuron density in the data with the empirical null distribution. A two-tailed *P*-value was calculated by integrating the probabilities of values in the null distribution that were as extreme as or more extreme than the observed value. To visualize the null distribution, we plotted it as a normalized histogram. In this representation, the height of each bar corresponds to the probability density of observing values in the respective range, such that the total area under the histogram integrates to one, while the height of each bar can take values larger than one. This normalization makes the histogram interpretable as an empirical probability distribution, rather than raw counts, which depend on the number of simulations and binning.

We used Prism (Graphpad, San Diego, USA) for standard statistical tests (ANOVA, Mann–Whitney and χ^2^ test), and custom Python code based on NumPy 1.21 to perform the statistical tests based on empirical null distributions. We considered significant *P* values below 0.05.

## Results

### Classification of grasping motor neurons according to activity and outcome

In the single-unit recording sessions, rats performed a total of 870 trials. Specifically, the first rat performed 177 trials (137 successes and 40 failures), the second rat 130 trials (63 successes and 67 failures), the third rat 148 trials (99 successes and 49 failures), the fourth rat 284 trials (204 successes and 80 failures) and the fifth rat 131 trials (106 successes and 25 failures).

From a behavioral perspective, we identified three main phases of limb movement during each trial, i.e. (i) from the paw detaching to its passing through the box aperture (1^st^ phase) and (ii) from the paw passing the box aperture to food touch (2^nd^ phase), and (iii) from food touch to the paw re-passing through the box aperture (3^rd^ phase). The first two phases were included in the pre-touch epoch, whereas the 3^rd^ phase was categorized within the post-touch epoch. The differences in duration between these phases during successful and failed grasping were not significant (1^st^ phase: U = 676.50, *P* = 0.4110; 2^nd^ phase: U = 585.00, *P* = 0.0750; 3^rd^ phase: U = 720.50.50, *P* = 0.7052; Fig. 1B).

We recorded from 78 neurons (15.6 ± 4.7 per rat). Among these neurons, 51% (40/78, 8.0 ± 2.8 for each rat) exhibited a consistent modulation in response to both success and failure (outcome-independent neurons; Fig. 1C and E), whereas 49% (38/78, 7.2 ± 2.0 per rat) displayed variations in their discharge profile when comparing the two outcomes (outcome-dependent neurons; Fig. 1D and F). For subsequent analyses, we focused on outcome-dependent, neurons that increased activity in successful trials, leaving out neurons firing only in failed trials, due to their low sample size (*n* = 3).

Outcome-independent neurons (Fig. 1G) were located mainly at coordinates corresponding to anterior and medial-middle parts of the forelimb area. Outcome-dependent neurons (Fig. 1H) did not exhibit any strong clustering within specific areas of the forelimb region, although they were predominantly observed in anterior and medial regions.

We classified each grasping neuron into two main populations, based on its discharge profile upon success and failure. Within these populations, we identified distinct neuronal subpopulations depending on whether the firing rate in one of the two touch-related epochs (pre- or post-touch) was significantly higher or comparable to the other touch-related epoch (Tables 1, S1 and S2). When defining the outcome-dependent subpopulations, we focused on neurons that increased activity in successful trials, excluding those modulated exclusively during failed trials, due their variability and low sample size (*n* = 3).

We identified three distinct subpopulations among the outcome-independent neurons. The first subpopulation (pre-touch dominant neurons; *n* = 14; Fig. 2A and Tables 1 and S1), showed a significant effect of epoch (F_1,52_ = 117.20, *P* < 0.0001), but not outcome (F_1,52_ = 1.25, *P* = 0.2685) or outcome × epoch interaction (F_1,52_ = 0.3511, *P* = 0.5561). Notably, Mann–Whitney tests revealed that activity was maximum in the pre-touch epoch, regardless of the grasping outcome (successful: U = 3.00, *P* < 0.0001; failed: U = 0.00, *P* < 0.0001). The second subpopulation (post-touch dominant neurons; *n* = 22; Fig. 2B and Tables 1 and S1) showed a significant effect of outcome (F_1,84_ = 6.20, *P* = 0.0148) and epoch (F_1,84_ = 243.90, *P* < 0.0001), but no outcome × epoch interaction (F_1,84_ = 1.39, *P* = 0.2420). Mann–Whitney tests indicate that this subpopulation increased maximally its discharge in the post-touch epoch for both successful (U = 5.00, *P* < 0.0001) and failed grasps (U = 10.00, *P* < 0.0001). Finally, the third subpopulation (epoch-neutral neurons; *n* = 4; Fig. 2C and Tables 1 and S1) showed a nonsignificant effect of outcome (F_1,12_ = 0.24, *P* = 0.6347), epoch (F_1,12_ = 3.11, *P* = 0.1032) or outcome × epoch interaction (F_1,12_ = 0.05, *P* = 0.8202). For these neurons, the activity was similar in both epochs for both outcomes. We found no difference in the amplitude of peak activity (Fig. 2D, top panel and Table S1) between successful and failed grasps for pre-touch dominant (U = 84.50, *P* = 0.5220) and epoch-neutral (U = 2.00, *P* = 0.1429) neurons, whereas we observed a difference for the post-touch dominant neurons (U = 158.50, *P* = 0.0320). Concerning the timing of peak activity (Fig. 2D, bottom panel, and Table S1), there were no significant differences between successful and failed grasps in all the three subpopulations, i.e. pre-touch dominant (U = 82.50, *P* = 0.4876), post-touch dominant (U = 173.00, *P* = 0.1054) and epoch-neutral (U = 6.00, *P* = 0.6571) neurons.

Among the outcome-dependent neurons, we further distinguished three subpopulations. The first subpopulation (success-pre-touch dominant; *n* = 11; Fig. 2E and Tables 1 and S2) showed a significant effect of epoch (F_1,40_ = 21.52, *P* < 0.0001) and outcome × epoch interaction (F_1,40_ = 6.25, *P* = 0.0166), but not outcome (F_1,40_ = 0.01, *P* = 0.9068). After conducting Bonferroni post-hoc testing, we discovered that this subpopulation exhibited the highest level of activity during the pre-touch epoch in successful trials, whereas in cases of failure, their activity remained consistent across the two epochs. The second subpopulation (success-post-touch dominant; *n* = 11; Fig. 2F and Tables 1 and S2) showed a significant effect of epoch (F_1,40_ = 14.36, *P* = 0.0005) and outcome (F_1,40_ = 5.26, *P* = 0.0272) but no outcome × epoch interaction (F_1,40_ = 3.46, *P* = 0.0704). Mann–Whitney tests revealed that these neurons showed the highest level of activity during the post-touch epoch in successful trials (U = 10.00, *P* < 0.0001), whereas firing rates remained similar in both epochs when the grasping task failed (U = 43.00, *P* = 0.2668). The third subpopulation (success-epoch-neutral; *n* = 13; Fig. 2G and Tables 1 and S2) showed a significant effect of outcome (F_1,48_ = 17.10, *P* = 0.0001), but not epoch (F_1,48_ = 1.87, *P* = 0.1777) or outcome × epoch interaction (F_1,48_ = 2.62, *P* = 0.1124). Mann–Whitney tests revealed that this class presented a similar activity during the two epochs in successful trials (U = 79.00, *P* = 0.7893) as well as when the grasping failed (U = 49.00, *P* = 0.0718). Regarding the amplitude of the peak activity (Fig. 2H, top panel and Table S2), we found a difference between successful and failed trials for all three subpopulations, i.e. success-pre-touch dominant (U = 26.00, *P* = 0.0150), success-post-touch dominant (U = 21.00, *P* = 0.0047) and success-epoch-neutral (U = 43.00, *P* = 0.0026) neurons. The timing of the peak activity (Fig. 2H, bottom panel and Table S2) remained consistent in the three subpopulations, namely success-pre-touch dominant (U = 48.00, *P* = 0.4259), success-post-touch dominant (U = 32.50, *P* = 0.0669) and success-epoch-neutral (U = 64.50, *P* = 0.3149) neurons.

### Outcome-independent and -dependent neurons across the forelimb movement representations

During the long-duration ICMS sessions, we stimulated 160 sites (32 sites per rat). Among these sites, 85% (136/160, 27.2 ± 0.4 for each rat) evoked forelimb movements. We did not consider the remaining 15% of sites (24/160, 4.8 ± 0.4 for each rat), since their stimulation did not induce any significant marker displacement. Among the forelimb-related sites, 37% (51/136, 10.2 ± 1.2 per rat) elicited only proximal movements, whereas 10% (13/136, 2.6 ± 1.4 per rat) elicited only distal movements. The remaining 53% of the forelimb-related sites (72/136, 14.4 ± 1.2 per rat) evoked a combination of proximal and distal movements.

After the classification of the evoked proximal movements, the resulting frequency distribution cortical maps revealed that abduction (Abd: x, 5.08 ± 0.99 mm; y, 21.30 ± 0.95 mm; z, 24.22 ± 1.33 mm) was mainly represented in the middle and posterior regions (*n* = 72; Fig. 3A), adduction (Add: x, −3.83 ± 1.92 mm; y, −9.70 ± 1.88 mm; z, 4.42 ± 2.23 mm) in the anterior region (*n* = 11; Fig. 3B), extension (Ext: x, 18.17 ± 1.21 mm; y, 8.82 ± 1.18 mm; z, 12.73 ± 1.61 mm) in the medial portion of the anterior region (*n* = 24; Fig. 3C), and retraction (Rtr: x, −10.13 ± 1.67 mm; y, 7.27 ± 0.99 mm; z, 17.01 ± 3.28 mm) in the medial posterior border (*n* = 3; Fig. 3D). We did not consider elevation (Elv: x, 1.17 ± 0.43 mm; y, 3.01 ± 0.39 mm; z, 10.92 ± 0.45 mm; *n* = 13), since it was scattered throughout M1. Considering distal movements, we found sites that elicited opening (Opn: x, 9.48 ± 0.49 mm; y, 4.64 ± 0.42 mm; z, 9.11 ± 0.30 mm) mainly in the middle and posterior regions (*n* = 56; Fig. 3E), closure (Clo: x, 12.94 ± 0.55 mm; y, 7.18 ± 1.29 mm; z, 10.80 ± 1.49 mm) in the anterior portion (*n* = 5; Fig. 3F), opening/closure sequence (Ocs: opening phase: x, 12.95 ± 0.56 mm; y, 4.56 ± 0.47 mm; z, 9.89 ± 0.61 mm; closing phase: x, 5.59 ± 0.95 mm; y, 4.56 ± 0.47 mm; z, 0.48 ± 0.41 mm) medially in the anterior regions (*n* = 11; Fig. 3G), and supination (Sup: x, 3.08 ± 1.02 mm; y, 4.82 ± 0.42 mm; z, 4.00 ± 0.26 mm) in the more lateral portion (*n* = 11; Fig. 3H).

We then compared the distribution maps of recorded neurons with those of proximal and distal evoked movements, to examine whether the proportion of neurons that were outcome-independent and outcome-dependent was overrepresented within each movement representation. Considering proximal movements (Fig. 4A), this proportion varied significantly across different movement representations (χ^2^₃ = 51.06, *P* < 0.0001, V_K_ = 0.3573). The outcome-dependent neuron density, defined as the number of outcome-dependent neurons per unit area, (Fig. 4B, top panels) in the actual data was within the bulk of the empirical null distribution obtained from random spatially uniform rearrangment of the neurons’ positions. Consequently, it was not significant for Abd (*P* = 0.5084), Add (*P* = 1.0000), Ext (*P* = 1.0000), and Rtr (*P* = 0.1105) representations. In contrast, the outcome-independent neuron density (Fig. 4B, bottom panels) was significantly higher than expected based on the empirical null distribution within a particular cortical region, i.e. the Ext representation (*P* = 0.0409). The density was somewhat elevated but not statistically significant within the Add representation (*P* = 0.0965), whereas clearly not significant within Abd (*P* = 0.2582) and Rtr (*P* = 1.0000). Turning to distal movements (Fig. 4C), we observed that the proportion of the outcome-independent and outcome-dependent neurons varied across movement representations (χ^2^_3_ = 20.89, *P* = 0.0001, V_K_ = 0.2285). The density of outcome-dependent neurons (Fig. 4D, top panels) was consistent with the empirical null distribution and, therefore, not significantly associated with Opn (*P* = 0.5234), Clo (*P* = 1.0000), Ocs (*P* = 0.5095), or Sup (*P* = 0.5115) representations. Instead, outcome-independent neurons (Fig. 4D, bottom panels) were significantly clustered within the Ocs representation (*P* = 0.0108), but showed no significant association with Opn (*P* = 0.1470), Clo (*P* = 0.3598), and Sup (*P* = 0.7377) representations.

## Discussion

In this study, we have investigated the discharge profile of grasping motor neurons and their distribution within the cortical movement map. First, we performed single-unit recordings in M1 while rats performed a grasping task. We isolated a number of responsive motor neurons, which we classified into two main populations: outcome-independent neurons, i.e. neurons maintaining the same discharge profile independent of the task’s successful or failed outcome, and outcome-dependent neurons, i.e. neurons presenting different activity profiles depending on the outcome. By considering the position relative to the recording grid of each neuron, we also constructed a cortical distribution map of these populations. Second, we compared the cortical distribution maps of these classes of motor neurons with those relative to complex forelimb movements evoked by ICMS on additional rats. The majority of outcome-independent neurons is localized within the forelimb extension (Fig. 4B) and paw open-closure (Fig. 4D) movement representations, whereas the outcome-dependent neurons are not clearly associated to a particular movement representation.

The activation of areas devoted to forelimb extension and paw opening-closure sequence, when appropriately combined, define the whole reach-grasp action (Bonazzi et al. 2013; Viaro et al. 2017). Consequently, we might conclude that neurons within motor representations directly involved in the execution of grasping, i.e. goal-directed actions, do not play a key role in error processing, as their modulation is primarily outcome-independent. On the other hand, our data is in agreement with previous ICMS experiments (Graziano et al. 2002; Ramanathan et al. 2006;Bonazzi et al. 2013 ; Viaro et al. 2017), showing that the motor cortex is arranged according to a map of coordinated movements within a behaviourally relevant frame, as opposed to a mere somatotopic organization based on muscles.

In contrast, outcome-dependent neurons are widely distributed across various movement representations. This lack of localization makes it impossible to link their activity to a specific step of the grasping movement as a whole. This finding may reflect the fact that errors can occur at any stage of the grasping task, or that error-related signals are relatively more abstract than the implementation details of the failed motor command. At the same time, it is important to consider that the spatial resolution of ICMS may be insufficient to resolve the fine-scale precision achievable by single unit recordings. While the stimulation of a single neuron can in principle evoke a movement or a behavioral response (Brecht et al. 2004; Houweling and Brecht 2008; Bernardi and Lindner 2017, 2019; Bernardi et al. 2021), ICMS is known to activate a significant number of neurons within the rodent cortex (Butovas and Schwarz 2003; Histed et al. 2009). Our data show that ICMS at a specific site reliably evokes movement. However, there is noticeable variability in each ICMS-evoked movement, which could result from a slightly different set of neurons being stimulated, as well as inter-subject variability. The spatial end-point variability observed in ICMS-evoked movements appears consistent with behavioral fluctuations that can lead to a failed grasp. Furthermore, maps derived from long-duration ICMS and those derived from single-neuron recording were obtained from two different sets of rats. Consequently, their comparison introduces a further potential inter-subject variability. Nevertheless, the dimensions of both the skull and brain do not change significantly over time in adult rats. By adhering to anatomical reference points (e.g. bregma), it is possible to translate cortical topography between animals of the same gender. Additionally, the ICMS technique has consistently revealed a stable topography of complex movement classes across the rat’s M1, along with a spatial topography corresponding to the locations towards which the paw is directed (Bonazzi et al. 2013; Viaro et al. 2017).

When exploring movement error it is important to delve into its components, which can emerge from two different mechanisms. The first relates to a problem occurring during motor planning while the other refers to the later component of motor execution. While the first reflects the erroneous selection of an action, given a specific context, execution errors emerge as the mismatch between the intended and effective action outcome. Execution errors are often investigated by applying an unpredictable alterations in the target’s location (i.e. commonly referred to as target errors; Diedrichsen et al. 2005). Here, execution errors consist in unsuccessful grasping attempts that are not induced by any experimental manipulation. These failures result from miscalibration of internal models and can be conceived a pure or spontaneous execution errors (i.e. no perturbation whatsoever is applied within the task).

Furthermore, it is relevant to consider the complexity of skilled grasping, which is composed of three successive phases, i.e. orientation, transport, and withdrawal, each one with its specificity (Alaverdashvili and Whishaw 2013). During the orientation phase, a rat uses olfactory cues, proprioception, whisker scanning, and tactile nose senses to locate food/object and determine the path to reach it with its paw (Whishaw and Tomie 1989; Alaverdashvili et al. 2008). Different levels of orientation perturbation do not prevent the ability of a rat to locate and grasp the pellet, albeit using a modified motor strategy (Parmiani et al. 2023). Transport and withdrawal are ballistic movements that involve lifting, reaching, and retraction of the paw (Whishaw and Karl 2014). All in all, the different phases make very different use of sensory feedback and implement very different motor control schemes. As a further influence, the magnitude of the potential reward and reward history are known to be tightly intertwined with the decision making processes regarding foraging behaviors to an extent that movement properties can vary dramatically in their vigor (Yoon et al. 2018). As a consequence, reward-driven arousal may affect the outcome of an individual’s performance (Watanabe et al. 2019) also in terms of error distribution and learning outcomes (Moskowitz et al. 2020). Here, we did not induce errors through distractions or target changes (Levy et al. 2020), nor did we manipulate sensory feedback or the reward scheme. Instead, we focused only on execution errors that are naturally emerging from overtrained actions. Consistently, in our task, the durations of the key steps did not differ between successful and failed outcomes.

Generally, ongoing movements can be rapidly adjusted (Goodale et al. 1986). The brain areas that play a major role in the real-time evaluation of discrepancies between the correct and the actual motor command are the cerebellum (Blakemore et al. 2001; Miall et al. 2001), the posterior parietal (Desmurget et al. 1999, 2001; Desmurget and Grafton 2000; Gréa et al. 2002) and medial frontal cortices (Botvinick et al. 2004; Ridderinkhof et al. 2004), and in particular the rostral cingulate zone (Picard and Strick 1996) and supplementary motor areas (Bonini et al. 2014). It has been previously shown that, when within-trial corrections do not prevent the movement’s failure, subsequent error signals indicate that goal was not achieved (Krigolson and Holroyd 2007). In this scenario, next-trial adjustments (Ridderinkhof et al. 2004; Ullsperger et al. 2014), possibly spanning several trials into the future (Shima and Tanji 1998; Quilodran et al. 2008), become relevant to optimize movements. In a recent study conducted with mice, success and failure of the previous trial differently modulated the layer V M1 neurons activity prior to the onset of the next trial, although this phenomenon did not lead to concomitant behavioral differences (Levy et al. 2020).

Here we show that outcome-independent neurons presented a similar discharge profile throughout the execution of the action regardless of whether the subject achieved the goal or made an error (Fig. 2A-C). A large share of outcome-independent neurons displayed their highest levels of activation during the pre-touch epoch, which suggests that these neurons played a role in encoding the movement pattern during the transport phase, paw shaping, and grip formation (Murata et al. 2000). Since rat M1 neurons exhibit visuomotor properties (Viaro et al. 2021), it is possible that the neuronal activity during various epochs is influenced by the modulation of action kinematics through online visual feedback (Churchill et al. 2000; Schettino et al. 2003; Winges et al. 2003; Rand et al. 2007). A similarly large share of outcome-independent neurons exhibited their maximum activation during the post-touch epoch. The post-touch dominance indicates that these neurons might be involved with after-touch events, such as retraction, limb/paw posture corrections and thus dependent on proprioceptive, tactile, and force feedback (Raos et al. 2006; Nelissen et al. 2011; Fadiga et al. 2013) to compute hand-position in the peripersonal space (Buneo and Andersen 2012). It is likely that epoch-neutral neurons exhibiting similar activity in both pre- and post-touch epochs, shared a combination of several aforementioned properties.

Among outcome-dependent neurons, which are tuned by the outcome, we classified neurons based on success outcome, grouping together neurons displaying a temporal shift of the maximum activity between the two outcomes and those showing an increased activity associated to only success. Neurons presenting a modulation only during failure were too few to be studied further, although they could be of great importance. As a fundamental concept, the difference in activity during the post-touch epoch of failed trials, with respect to that observed in successful trials, likely reflects the absence of grip-related sensorimotor feedback following a failed prehension (Raos et al. 2006; Nelissen et al. 2011). Conversely, the two subpopulations classified as success-pre-touch dominant and success-epoch-neutral (Fig. 2E, G and H), which displayed a significant firing during the pre-touch epoch, may be associated with an anomaly in motor programming and/or motor output before touch (Murata et al. 2000). Notably, nearly all outcome-dependent neurons exhibited a discharge pattern that was asymmetric with respect to touch time. Alterations in temporal asymmetry have been linked to various neuropathologies affecting both motor and higher cognitive functions (Schindler et al. 2016; Zanin et al. 2020; Bernardi et al. 2023; De La Fuente et al. 2023) and may reflect impairments in error feedback circuits. Another important observation regarding success-pre-touch dominant neurons is that activity differences between correct and error trials cannot be attributable to error feedback processing (Watanabe et al. 2019) because errors have yet to occur. Therefore, these differences may contribute to feedforward error processing and thus serve as a predictive marker associated with potential errors. Activity predicting the success or failure of the subject has been observed in the dorsolateral prefrontal cortex using functional magnetic resonance imaging in humans during the period before actual responses (Hester et al. 2004). Also the cingulate and pre-supplementary motor area seem to serve a feedforward function during motor tasks, in particular to correct for movement errors in a proactive manner (Isoda and Hikosaka 2007). These areas are also the most likely generators of human Error-Related Negativity (ERN), which is elicited by error feedback (Falkenstein et al. 1995; Gehring et al. 2018). ERN was later found to anticipate errors, thus potentially also reflecting a feedforward component (Krigolson and Holroyd 2007). The presence of a feedforward error component encoded M1 single units does not support a purely accessory role of primary motor areas in error processing. Rather, this should be taken as preliminary evidence that M1 contributes to error processing.

This fact, may have important implication for brain-machine interfaces (BMIs). Current BMIs decode neural correlates of movement intention from the motor cortex, as ascertained in animal models (Taylor et al. 2002; Carmena et al. 2003; Musallam et al. 2004; Santhanam et al. 2006; Moritz et al. 2008; Velliste et al. 2008; Ganguly and Carmena 2009; Ethier et al. 2012; Gilja et al. 2012; Kao et al. 2015; Kao et al. 2016; Nuyujukian et al. 2017) and in humans (Hochberg et al. 2006; Hochberg et al. 2012; Collinger et al. 2013; Aflalo et al. 2015; Gilja et al. 2015; Jarosiewicz et al. 2015; Wodlinger et al. 2015; Bouton et al. 2016; Pandarinath et al. 2017; Biasiucci et al. 2018; Flesher et al. 2021). However, BMI users have not yet achieved consistently optimal movement kinematics, and errors remain a persistent challenge (Fidêncio et al. 2022). Errors usually require the BMI user to take timely corrective actions, which may be possible in an optimally-designed lab system, but impractical in real-world situations (Even et al. 2017). Gaining a better understanding of real-time error signals within the same cortical area where movement intentions are decoded could be crucial to enhance the effectiveness of BMIs. In this vein, being able to identify outcome-dependent signals that, already in the planning or early execution stages, inform about the likelihood of a motor error might allow far more effective and timely adaptation to the contextual situation. Furthermore, the fact that such outcome-dependent neurons did not show a clear localization within the M1 somatotopy suggests that BMIs need to draw input from a broader region of the motor cortex to attain control over complex movements and, potentially, to extract information that is not strictly related to motor execution but may have great potential for feedforward error processing.

## Supplementary Material

Supplementals_materials_Accepted_bhaf032

## Data Availability

Raw data were generated at the Department of Neuroscience and Rehabilitation of the University of Ferrara. Derived data supporting the findings of this study are available from the corresponding author on request.

## Abbreviations

Primary motor cortex, M1; Intracortical Microstimulation, ICMS); Mediolateral, ML; Anteroposterior, AP; Abduction, Abd; Adduction, Add; Extension, Ext; Retraction, Rtr; Opening, Opn; Closure, Clo; Opening/closure sequence, Ocs; Supination, Sup.
