# Supplementary material for: Differential motor neuron activity in rats during successful and failed grasping
Source: Cereb Cortex. 2025 Mar 2;35(2):bhaf032. doi: 10.1093/cercor/bhaf032 (PMC12448896; doi:10.1093/cercor/bhaf032)
Supplement: Supplementals_materials_Accepted_bhaf032 [file supplementals_materials_accepted_bhaf032.pdf]

Supplementary Materials

Differential motor neuron activity in rats during successful and failed grasping

Riccardo Viaro,[a,b,*] Davide Bernardi,[b,c] Emma Maggiolini,[a] Alessandro D'Ausilio,[a,b] Carolina Giulia Ferroni,[b] Pierantonio Parmiani,[a] and Luciano Fadiga[a,b]

[a]*Section of Physiology, Department of Neuroscience and Rehabilitation, University of Ferrara, 44121, Ferrara, Italy;* [b]*Center for Translational Neurophysiology, Istituto Italiano di Tecnologia, 44121, Ferrara, Italy;* [c]*Department of Physics and Astronomy, University of Padova, 35131, Padova, Italy*

***Corresponding author:**

Riccardo Viaro, PhD

Section of Physiology,

Department of Neuroscience and Rehabilitation,

University of Ferrara,

via Fossato di Mortara 17-19,

44121 Ferrara, Italy

Phone: +39 0532 455243

E-mail: riccardo.viaro@unife.it

ORCID: 0000-0002-8384-1440

	Success	Failure	Statistics
**Pre-touch dominant neurons** **n = 14**			Outcome: $F_{1,52}$=1.25, p=0.2685<br>**Epoch: $F_{1,52}$=117.20, p<0.0001**<br>Outcome×Epoch: $F_{1,52}$=0.3511, p=0.5561
**Averaged activity**			
Pre-touch epoch	0.72±0.04	0.74±0.03	U=94.00, p=0.8627
Post touch epoch	0.19±0.06	0.27±0.05	U=70.00, p=0.2078
**Peak activity**			
Amplitude	0.95±0.02	0.91±0.03	U=84.50, p=0.5220
Timing	-227.10±18.35	-205.70±20.80	U=82.50, p=0.4876
**Post-touch dominant neurons** **n = 22**			**Outcome: $F_{1,84}$=6.20, p=0.0148**<br>**Epoch: $F_{1,84}$=243.90, p<0.0001**<br>Outcome×Epoch: $F_{1,84}$=1.39, p=0.2420
**Averaged activity**			
Pre-touch epoch	0.27±0.03	0.23±0.04	U=222.5, p=0.6547
Post touch epoch	0.79±0.03	0.68±0.03	**U=120.5, p=0.0037**
**Peak activity**			
Amplitude	0.94±0.03	0.83±0.04	**U=158.50, p=0.0320**
Timing	166.40±16.87	123.60±14.33	U=173.00, p=0.1054
**Epoch-neutral neurons** **n = 4**			Outcome: $F_{1,12}$=0.24, p=0.6347<br>Epoch: $F_{1,12}$=3.11, p=0.1032<br>Outcome×Epoch: $F_{1,12}$=0.05, p=0.8202
**Averaged activity**			
Pre-touch epoch	0.58±0.04	0.62±0.05	U=5.50, p=0.5429
Post touch epoch	0.68±0.05	0.69±0.05	U=7.50, p=0.9714
**Peak activity**			
Amplitude	0.88±0.07	1.00±0.00	U=2.00, p=0.1429
Timing	-25.00±27.54	55.00±80.57	U=6.00, p=0.6571

Table S1. Subpopulations of the outcome-independent neurons in successful and failed grasping trials. For each subpopulation, the table presents the number of neurons, averaged activity in the pre-touch and post touch epochs, amplitude and timing of the peak activity. Statistics refer to the overall ANOVA results and Mann-Whitney specific results. Averaged and peak activities are expressed in net normalized activity. Timing is expressed in ms. Significant values are highlighted in bold.

	Success	Failure	Statistics
**Success-pre-touch dominant neurons** **n = 11**			Outcome: $F_{1,40}$=0.01, p=0.9068 **Epoch: $F_{1,40}$=21.52, p<0.0001** **Outcome×Epoch: $F_{1,40}$=6.25, p=0.0166**
**Averaged activity**			
Pre-touch epoch	0.72±0.05	0.52±0.07	**U=29.00, p=0.0385**
Post touch epoch	0.12±0.10	0.34±0.10	U=36.00, p=0.1155
**Peak activity**			
Amplitude	0.92±0.06	0.76±0.07	**U=26.00, p=0.0150**
Timing	-187.30±22.93	-136.40±47.47	U=48.00, p=0.4259
**Success-post-touch dominant neurons** **n = 11**			**Outcome: $F_{1,40}$=5.26, p=0.0272** **Epoch: $F_{1,40}$=14.36, p=0.0005** Outcome×Epoch: $F_{1,40}$=3.46, p=0.0704
**Averaged activity**			
Pre-touch epoch	0.36±0.06	0.33±0.09	U=59.00, p=0.9313
Post touch epoch	0.79±0.06	0.47±0.09	**U=16.00, p=0.0024**
**Peak activity**			
Amplitude	0.94±0.05	0.66±0.08	**U=21.00, p=0.0047**
Timing	154.50±27.11	63.64±43.80	U=32.50, p=0.0669
**Success-epoch-neutral neurons** **n = 13**			**Outcome: $F_{1,48}$=17.10, p=0.0001** Epoch: $F_{1,48}$=1.87, p=0.1777 Outcome×Epoch: $F_{1,48}$=2.62, p=0.1124
**Averaged activity**			
Pre-touch epoch	0.67±0.06	0.50±0.08	**U=46.00, p=0.0499**
Post touch epoch	0.69±0.05	0.30±0.07	**U=21.00, p=0.0006**
**Peak activity**			
Amplitude	0.90±0.06	0.73±0.08	**U=43.00, p=0.0026**
Timing	-1.54±28.24	-41.54±37.92	U=64.50, p=0.3149

Table S2. Subpopulations of the outcome-dependent neurons in successful and failed grasping trials. For each subpopulation, the table presents the number of neurons, averaged activity in the pre-touch and post touch epochs, amplitude and timing of the peak activity. Statistics refer to the overall ANOVA results and Mann-Whitney specific results. Averaged and peak activities are expressed in net normalized activity. Timing is expressed in ms. Significant values are highlighted in bold.
